# Hydroxymethylated Cytosines Are Associated with Elevated C to G Transversion Rates

**DOI:** 10.1371/journal.pgen.1004585

**Published:** 2014-09-11

**Authors:** Fran Supek, Ben Lehner, Petra Hajkova, Tobias Warnecke

**Affiliations:** 1 EMBL-CRG Systems Biology Unit, Centre for Genomic Regulation (CRG), Barcelona, Spain; 2 Universitat Pompeu Fabra (UPF), Barcelona, Spain; 3 Division of Electronics, Rudjer Boskovic Institute, Zagreb, Croatia; 4 Institució Catalana de Recerca i Estudis Avançats, Centre for Genomic Regulation (CRG) and UPF, Barcelona, Spain; 5 Reprogramming and Chromatin Group, MRC Clinical Sciences Centre, Imperial College, Hammersmith Campus, London, United Kingdom; 6 Molecular Systems Group, MRC Clinical Sciences Centre, Imperial College, Hammersmith Campus, London, United Kingdom; Université Claude Bernard - Lyon 1, France

## Abstract

It has long been known that methylated cytosines deaminate at higher rates than unmodified cytosines and constitute mutational hotspots in mammalian genomes. The repertoire of naturally occurring cytosine modifications, however, extends beyond 5-methylcytosine to include its oxidation derivatives, notably 5-hydroxymethylcytosine. The effects of these modifications on sequence evolution are unknown. Here, we combine base-resolution maps of methyl- and hydroxymethylcytosine in human and mouse with population genomic, divergence and somatic mutation data to show that hydroxymethylated and methylated cytosines show distinct patterns of variation and evolution. Surprisingly, hydroxymethylated sites are consistently associated with elevated C to G transversion rates at the level of segregating polymorphisms, fixed substitutions, and somatic mutations in tumors. Controlling for multiple potential confounders, we find derived C to G SNPs to be 1.43-fold (1.22-fold) more common at hydroxymethylated sites compared to methylated sites in human (mouse). Increased C to G rates are evident across diverse functional and sequence contexts and, in cancer genomes, correlate with the expression of Tet enzymes and specific components of the mismatch repair pathway (MSH2, MSH6, and MBD4). Based on these and other observations we suggest that hydroxymethylation is associated with a distinct mutational burden and that the mismatch repair pathway is implicated in causing elevated transversion rates at hydroxymethylated cytosines.

## Author Summary

Most cytosines that occur in a CpG context in mammalian genomes are methylated. Methylation has important functional consequences in the cell but also affects genome evolution. Notably, methylated cytosines are prone to deaminate and constitute mutational hotspots in mammalian genomes. Recently, a series of other modifications, derived from the oxidation of methylated cytosines, was shown to exist in various mammalian cell types including embryonic stem cells. The most abundant of these modifications is 5-hydroxymethylcytosine. In this work, we ask whether methylated and hydroxymethylated cytosines are subject to the same mutational biases or lead to distinct patterns of genome evolution. To do so, we examine differences between individuals, between species, and between normal and cancer tissues alongside high-resolution maps of DNA methylation and hydroxymethylation in the human and mouse genomes. Unexpectedly, we find that hydroxymethylated cytosines are associated with more cytosine to guanine changes in both human and mouse populations, in closely related species, and in the context of somatic evolution in tumors. Based on multiple lines of evidence, we suggest that the different patterns of sequence evolution at methylated and hydroxymethylated sites are owing to differences in how these sites are handled by the DNA repair machinery.

## Introduction

In mammalian genomes, most cytosines that occur in a CpG context are methylated. 5-methylcytosines (5mCs) at CpG dinucleotides exhibit mutation rates an order of magnitude above that of unmodified cytosines, a consequence both of their greater propensity to deaminate and error-prone repair of the resulting thymine [1]. This mutational liability is evident in higher levels of single nucleotide polymorphisms (SNPs) segregating at CpGs in mammalian populations [2]–[4], higher rates of divergence between species at these sites [5], [6], and higher somatic mutation rates in many cancer genomes compared to other nucleotide contexts [7].

Recently, it has become clear that the repertoire of naturally occurring cytosine modifications in mammals extends beyond 5mC to include a series of modifications derived from successive rounds of 5mC oxidation: 5-hydroxymethylcytosine (5hmC), 5-formylcytosine (5fC), and 5-carboxylcytosine (5caC) [8], [9]. 5fC and 5caC have been found to occur at low frequencies in genome-wide studies in human and mouse (∼0.01–0.0001% of cytosines [10]), consistent with being rapidly converted intermediates in an active demethylation pathway that involves cumulative oxidation of 5mC by Tet enzymes and the eventual removal of 5fC or 5caC via base excision repair (BER) [11]. In contrast, 5hmC has been detected at relatively high levels (∼0.1% of cytosines) in certain cell types including Purkinje cells, embryonic stem (ES) cells and primordial germ cells, suggesting that it might be present as a quasi-stable epigenetic mark rather than merely a transient demethylation intermediate [12].

In the context of the high mutational burden of 5mC and considering that 5hmC can be present as a stable epigenetic mark, we wondered whether methylated and hydroxymethylated sites might be associated with distinct patterns of sequence evolution, perhaps as a consequence of divergent mutational biases. For example, in mammalian systems, repair of 5hmU:G mismatches (derived from 5hmC deamination) by the glycosylases TDG and SMUG1 is less error-prone than dealing with 5mC-derived T:G mispairs [13]. As a consequence, residues that spend a significant proportion of their lifetime in the germline in a 5hmC state might be less mutagenic than 5mC sites.

Here, to elucidate the evolutionary repercussions of hydroxymethylation, we integrate population genomic, inter-species divergence and somatic mutation data from tumors with publicly available base-resolution maps of 5hmC and 5mC in human and mouse ES cells [14]–[17]. As further discussed below, 5hmC profiles in ES cells show similarities to 5hmC profiles at different stages of germline development, making ES cells a relevant model system to investigate the impact of hydroxymethylation on sequence evolution.

## Results

DNA methylation at single nucleotide resolution is commonly detected using bisulfite sequencing. While bisulfite sequencing provides a high-coverage view of methylation across the genome, it does not discriminate between 5hmC and 5mC modifications: any site identified as modified by bisulfite sequencing could be 5hmC or 5mC. However, alternative sequencing strategies, notably Tet-assisted bisulfite sequencing (TAB-Seq) [15] and oxidative bisulfite sequencing [18], can, when used in conjunction with traditional bisulfite sequencing, specifically identify 5hmC residues. We therefore defined methylated, hydroxymethylated, and unmethylated cytosines as follows: for human ES cells (H1 hESC), we followed the binary classification into methylated and unmethylated cytosines provided by [16]. For mouse embryonic stem cells (E14TG2a mESC), to make results more comparable between species, we decided to emulate these binary calls. To do so, we examined how the H1 hESC binary classification relates to the underlying quantitative read data (Figure S1). Sites where less than 20% of the reads support methylation are typically classified as unmethylated. Accordingly, we classified mouse cytosines as unmethylated, if the fraction of reads supporting methylation in mESC [14] was lower than 0.2. For both human and mouse, we then subdivided the methylated class into 5hmC and 5mC sites, with sites classified as 5hmC if at least one read from TAB-Seq in the same cell line [15] supported hydroxymethylation. This simple categorization allows us to contrast patterns of sequence evolution associated with different methylation states. In reality, the three states coincide at a given cytosine across a population of cells and indeed across the life cycle of the cytosine, with 5mC a necessary precursor to generate 5hmC.

### Elevated C to G transversion rates at hydroxymethylated sites

**Figure 1:**
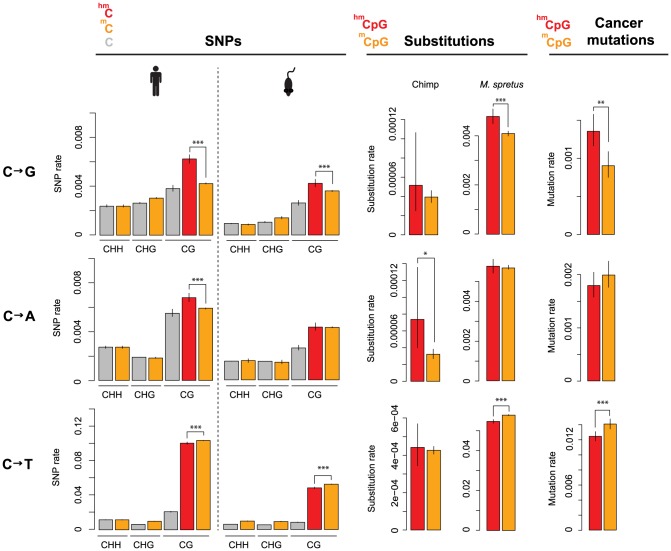
Evolutionary rates differ according to methylation state. Rates of cytosine loss are given as a function of methylation status (5hmC: red; 5mC: orange; C: grey), methylation context (CHH, CHG, CG; H = A/C/T) and evolutionary event (derived SNPs in human or mouse population; substitutions along the chimp or *M. spretus* lineage; somatic mutations in cancer genomes). Only significant differences between 5hmC and 5mC sites in a CpG context are highlighted (***P<0.001; **P<0.01; *P<0.05). Error bars are 95% confidence intervals, calculated using Wilson's interval score for single proportions.

We then focussed on residues located outside of repeat regions, covered by at least ten sequencing reads in the pertinent bisulfite experiment, and amenable to accurate SNP calling (Materials and Methods). Further, as cell line genotypes differ from the reference genomes, we confined analysis to sites with known cell line genotype, using ENCODE short read data to genotype H1 hESC (Materials and Methods). For this high-confidence dataset, we asked what fraction of 5mC, 5hmC, and C sites are associated with a derived SNP (“SNP rate”) in the human population and across 17 different laboratory or wild-derived inbred mouse strains. As shown in Figure 1, there is a small (but significant) reduction in C to T SNP rates at 5hmC compared to 5mC sites, consistent with less error-prone repair of 5hmU compared to T as suggested above. Unexpectedly, however, 5hmC sites in both human and mouse exhibit substantially higher rates of C to G transversions than 5mC sites, with C to A rates additionally elevated in human. Regarding the relative frequency of different base changes, transitions are an order of magnitude more common than transversions for both 5mC and 5hmC, likely reflecting high mutation rates following deamination.

Next, we considered rate differences at the level of divergence between species. For sites inferred to be cytosines in the human-chimp ancestral genome (see Materials and Methods), we examined substitutions along the chimp lineage as a function of methylation state in human. Consistent with the population genomic data, transversion rates are higher at 5hmC sites (Figure 1). Analysis of substitutions in the *M. spretus* genome – relative to the *M. musculus-M. spretus* ancestral genome and *M. musculus* methylation state – echoes this result: C to G rates are higher at 5hmC sites than at 5mC sites (Figure 1).

### Different evolutionary regimes at hydroxymethylated sites are independent of sequence and functional context

The incidence of 5hmC sites varies according to regional GC content [15], [19], functional context (intron, exon, promoter, etc.) [20], and chromatin environment, where it is associated with active transcription and certain enhancer states [21], [22]. 5mC and 5hmC sites might therefore exhibit distinct patterns of sequence change not because of intrinsic (mutational) differences between the two marks but because they are unevenly represented in functional elements or genomic regions that are governed by disparate mutational and/or selective regimes [23], [24]. Indeed, examining derived allele frequencies (DAFs) in the human population we find a significant excess of rare alleles at 5mC compared to 5hmC sites (P<10^−20^), suggesting stronger average purifying selection at 5mC sites (Figure S2).

In order to isolate 5hmC/5mC-specific patterns of evolution that are independent of functional context and therefore likely mutational in nature, we adopted the following strategy: for every 5hmC site we selected a 5mC site that matches the 5hmC site with regard to local (±50 nt around the focal site) and regional (±500 nt) GC content, chromatin state, biotype, the upstream neighbouring nucleotide and the methylation level of the focal cytosine (see Materials and Methods and Table S1 for details). Matching for methylation level is particularly important given previous findings that more highly methylated CpGs in human sperm are associated with a greater frequency of rare derived alleles [4], consistent with selection being stronger, on average, at highly methylated sites.

Concurrently matching across multiple criteria in this fashion is feasible because 5mC sites vastly outnumber 5hmC sites so that a match can be found for a large fraction of 5hmCs. We did not include unmethylated cytosines in this analysis because matching across three categories severely reduces sample size. As mammalian hydroxymethylation occurs almost exclusively at CpG dinucleotides [15], [20], we focus on sites in the CpG context. All rate estimates below, including in the context of tumor evolution, refer to this context.

This matching procedure yields 121604 and 154060 5hmC-5mC pairs for human and mouse, respectively, which are matched with regard to various potential confounders and no longer differ significantly in their DAF spectra (P = 0.1, Figure S2), suggesting a similar distribution of selective constraints for the two classes of sites.

**Figure 2:**
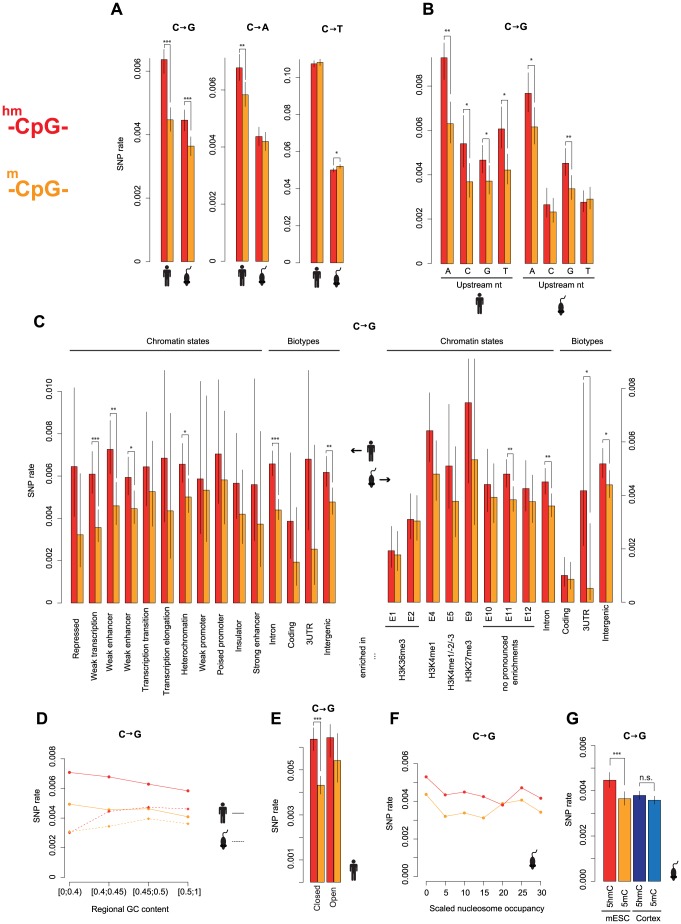
Elevated C to G rates at 5hmC sites across different sequence and functional contexts. (A) Genome-wide rates of cytosine loss at matched 5hmC and 5mC sites in the human and mouse population. (B–F) Elevated C to G SNP rates are evident for different upstream neighbouring nucleotides (B), chromatin states and biotypes (C), regional GC content (±500 nt around the focal site) (D), open and closed chromatin (E), and different levels of nucleosome occupancy (scaled nucleosome occupancy as defined in [62]) (F). (G) Neuron-specific 5hmC sites derived from frontal cortex of adult mice are compared to matched 5mC sites and presented side by side with ESC matched sites (same as in Figure 2A) (***P<0.001; **P<0.01; *P<0.05). Error bars are 95% confidence intervals, calculated using Wilson's interval score for single proportions.

Comparing SNP rates across matched sites suggests that differences in C to T rates between 5mC and 5hmC sites are indeed minor, and only remain marginally supported in mouse (Figure 2A, fold difference in rate (5hm/5mc): human: 0.99; mouse: 0.96). Importantly, however, pronounced differences in C to G transversion rates remain evident in both mouse and human (fold difference: human: 1.43; mouse: 1.22). Moreover, faster C to G rates at 5hmC sites are found across different chromatin states, biotypes (Figure 2C, Human: P<8*10^−6^, Mouse: P<0.0005; binomial test, testing for likelihood of all chromatin states showing enrichment in the same direction), and GC content levels (Figure 2D) and appear independent of the immediate nucleotide context (Figure 2B). For many of these subsets, differences are individually significant and we do not find a single context where the C to G rate is faster at 5mC sites. Furthermore, the effect is insensitive to nucleosome occupancy (Figure 2F) and observed in both open and closed chromatin as defined by the ENCODE project for H1 hESC (Figure 2E), suggesting that it is not simply a corollary of differential DNA accessibility, with, for example, more open chromatin structure facilitating Tet-mediated 5hmC generation [25] but also rendering DNA more prone to oxidative damage, a cause of C to G transversions [26].

### Embryonic stem cells provide adequate models to assess the evolutionary repercussions of hydroxymethylation

Having systematically accounted for differences in functional and sequence context, we reasoned that differences between 5mC and 5hmC sites likely reflect mutational biases. However, any mutational bias model rests on the assumption that (hydroxy)methylation patterns in ES cells are predictive of patterns in the germline and can therefore contribute mechanistically to a 5hmC-related mutation signature. To evaluate this assumption we first considered base resolution 5hmC maps for mouse neurons (adult frontal cortex) [17]. In particular, we focused on sites with evidence for hydroxymethylation in neurons but *not* in ES cells. Hydroxymethylation that is present exclusively in differentiated cells such as frontal cortex neurons should have no bearing on mutation dynamics in the germline. Neuron-specific 5hmC sites should, in mutational terms, behave like germline 5mC sites. We repeated the matching procedure described above, but now pairing neuron-specific 5hmC sites to sites called as 5mC in both ES cells and neurons. As predicted, there is no difference in C to G rates between the matched pairs (Figure 2G) and rates at neuron-specific 5hmC sites are significantly lower than at 5hmC sites in mESCs (P = 0.0009). Importantly, hydroxymethylation is more common in neurons, so this result is not an artefact of reduced power (number of matched pairs N = 428032).

The genomic incidence of hydroxymethylation has previously been examined for different stages of mouse spermatogenesis, using a chemical labelling method followed by enrichment and sequencing [27]. We find that 5hmC sites in ES cells are overrepresented in 5hmC-enriched regions in sperm, particularly at earlier stages of spermatogenesis (Figure S3). In addition, at multiple stages of spermatogenesis we find significant differences in C to G SNP rates (calculated for 5hmC and 5mC sites in ES cells) in 5hmC-enriched regions (Figure S3). In contrast, we never observe significant differences in regions without 5hmC enrichment. Note that there is high overlap in 5hmC-enriched regions across different stages of spermatogenesis [27], precluding statistically meaningful analysis of sites exclusively hydroxymethylated at some stages but not others. Future base resolution data will be required to establish more precisely to what degree hydroxymethylation patterns in the germline and ES cells overlap. However, based on the data presented and unpublished data showing high levels of similarity between 5hmC profiles in ES cells and the early germline (P. Hajkova, unpublished results), we suggest that ES cells constitute a relevant proxy to study the evolutionary repercussions of hydroxymethylation.

### Hydroxymethylation quantitatively predicts C to G transversion rates in humans

**Figure 3:**
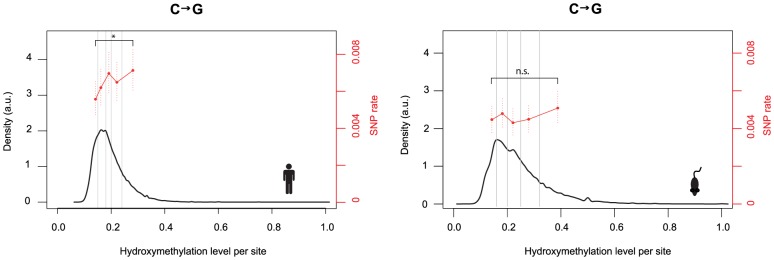
Hydroxmethylation levels correlate with C to G rates. Density plots depict the distribution of hydroxymethylation levels at 5hmC sites for the human and mouse genome. To calculate C to G SNP rates as a function of hydroxymethylation levels (% reads supporting hydroxymethylation at a given cytosine in [15]), cytosines were assigned to bins (demarcated by vertical lines) according to their hydroxymethylation levels and a single rate estimate was derived for each bin. Bin sizes were chosen so that each bin contains the same number of C to G changes (N = 155 for human, N = 138 for mouse). C to G SNP rates were then compared for the terminal bins (*P<0.05). Error bars are 95% confidence intervals, calculated using Wilson's interval score for single proportions.

We reasoned that – if elevated C to G rates are mechanistically linked to hydroxymethylation – they might be higher at sites where the 5hmC mark is more prevalent. Hydroxymethylation is non-stoichiometric and sites classified as 5hmC are typically hydroxymethylated in a minority of cells in the population. We therefore tested whether cytosines with higher levels of hydroxymethylation exhibit higher SNP rates. This is indeed the case in human (Figure 3, P = 0.04; test of proportions comparing terminal bins). Although an increase towards higher rates for highly hydroxymethylated sites is also apparent in mouse, the difference is not significant.

### 5hmC sites are associated with higher C to G transversion rates in cancer genomes

If differences at 5hmC sites reflect mutational biases, such biases might also operate in the context of somatic evolution. To explore this possibility, we compiled a catalogue of single nucleotide mutations across 346 diverse fully sequenced cancer genomes (see Materials and Methods) and compared somatic mutation rates for the set of matched 5hmC and 5mC sites described above. Again, we find significantly elevated C to G rates at 5hmC sites (Figure 1).

**Figure 4:**
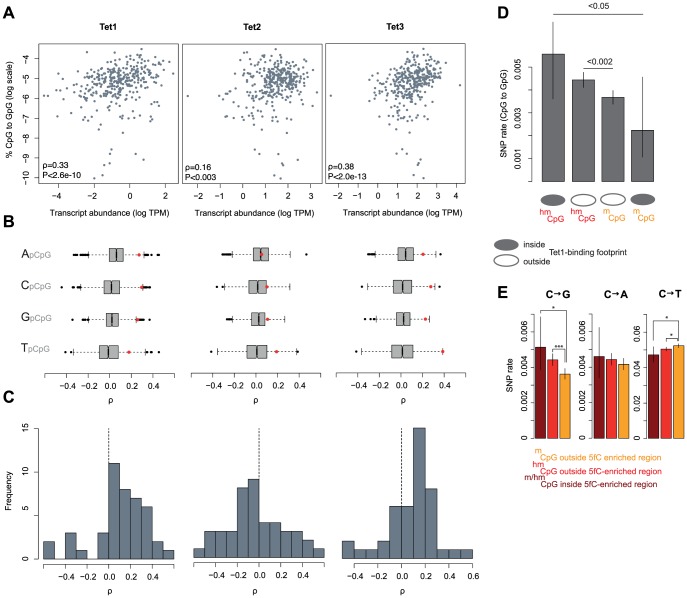
The expression and binding of Tet enzymes correlates with C to G rates. (A) Expression levels of Tet1 and Tet3, but not Tet2, correlate with C to G somatic mutation rates across 346 cancer genomes. (B) For different upstream contexts, correlation coefficients (Spearman's rho) between C to G rates and expression levels were computed for the three Tet genes (red dots) and their respective set of control genes (grey/black, see main text). Whiskers extend to approximately 1.5*IQR (interquartile range) below/above the bottom/top quartile of the data, (see R documentation [69] for details). (C) Distribution of correlation coefficients (C to G rates ∼ Tet expression) calculated independently for 44 different cancer type-upstream context combinations. (D) C to G SNP rates at 5hmC and 5mC sites as a function of Tet1-binding in mESCs. (E) Rates of cytosine loss for the matched set of sites as a function of location inside or outside 5fC-enriched regions in mESC as defined by [32]. Error bars are 95% confidence intervals, calculated using Wilson's interval score for single proportions. TPM: transcripts per million.

We then examined the relationship between C to G rates in tumors and the expression of Tet proteins. Tet proteins catalyse the oxidation of 5mC to 5hmC and therefore constitute a critical rate-limiting step for 5hmC generation, as evident in lower genome-wide levels of 5hmC in mouse ES cells where Tet1/2 protein levels are diminished following shRNA-mediated knock-down [28]. As Tet expression levels affect the relative abundance of 5hmC, we predict that Tet expression should positively correlate with C to G mutation rates, irrespective of low baseline hydroxymethylation levels in cancer cells compared to ES cells or neurons. Figure 4A highlights that, considering mutations across 346 cancer genomes, there are positive correlations between the proportion of all mutations that are C to G (%C2G) and the transcript levels of Tet1 and Tet3. To ascertain whether correlations are stronger than expected by chance, we compared each Tet gene to a bespoke control set of ∼1500 genes most similar in median expression and dispersion across tumors (see Materials and Methods). As some mutational processes that operate in cancer genomes are known to exhibit nucleotide context biases [7], we present correlation coefficients separately for each upstream neighbouring nucleotide. The results confirm that expression levels for Tet1 and Tet3, but not Tet2, are strongly associated with %C2G (Figure 4B, Tet1: P<1.57*10^−05^; Tet2: P>0.05; Tet3: P<4.58*10^−07^; Stouffer test combining P values across contexts) and largely insensitive to upstream nucleotide context, suggesting that we are not dealing with a known, context-dependent mutational process.

Considering correlations separately for 11 different types of cancer (colorectal cancers, breast cancers, etc.; see Table S2 for a complete list), we also predominantly observe positive correlations for Tet1 (35 out of 44 cancer type-context combinations) and Tet3 (32/44) but not for Tet2 (17/44, Figure 4C). In terms of the variance explained by Tet expression levels, correlations are comparable in magnitude to the correlation between APOBEC signature mutations and APOBEC expression recently reported for breast cancer genomes [29].

To probe further into the putative link between Tet activity, hydroxymethylation, and C to G transversions, we considered SNP rates in relation to Tet1 binding footprints, determined on a genome-wide scale in mouse ES cells [30]. Although coinciding surprisingly poorly with the distribution of 5hmC sites [30], [31], we reasoned that Tet1 binding can be exploited as a sentinel for intrinsic hydroxymethylation risk alongside 5hmC/5mC status itself. 5mC sites can be seen as refractory to hydroxymethylation if they are located inside a Tet1 binding footprint yet fail to show signs of hydroxymethylation. Conversely, 5hmC residues located in Tet1 binding footprints clearly *can* be hydroxymethylated and are likely hydroxymethylated more reproducibly across cells and time given the presence of Tet1. On average, 5hmC sites inside Tet1 binding footprints should therefore spend more time in a hydroxymethylated state than 5hmC sites outside footprints. In line with this scenario, we observe the highest and lowest rate of C to G transversions at 5hmC and 5mC sites inside Tet1 binding footprints, respectively (Figure 4D). This finding also argues against a scenario where elevated transversion rates are simply the consequence of a locally elevated non-specific oxidation risk associated with the presence of Tet proteins.

If different mutational dynamics at 5hmC sites are associated with Tet-mediated oxidation, we might also suspect regions of high 5hmC turnover – where 5hmC is frequently further oxidized to 5fC/5caC and eventually undergoes BER – to show more pronounced rate differences. Considering the presence of 5fC as an indicator of high 5hmC turnover, we compared SNP rates inside and outside regions found to be enriched for 5fC in mESC [32]. We observe trends in the expected direction for all base changes, with C to G rates more pronounced for sites located in 5fC-enriched regions (Figure 4E). However, because there are few 5fC-enriched regions and therefore few nucleotides available for analysis, SNP rate estimates are correspondingly noisy, likely precluding the detection of a significant differences between 5hmC sites and residues located in 5fC-enriched regions.

### Higher rates of cytosine loss at asymmetrically hydroxymethylated sites

**Figure 5:**
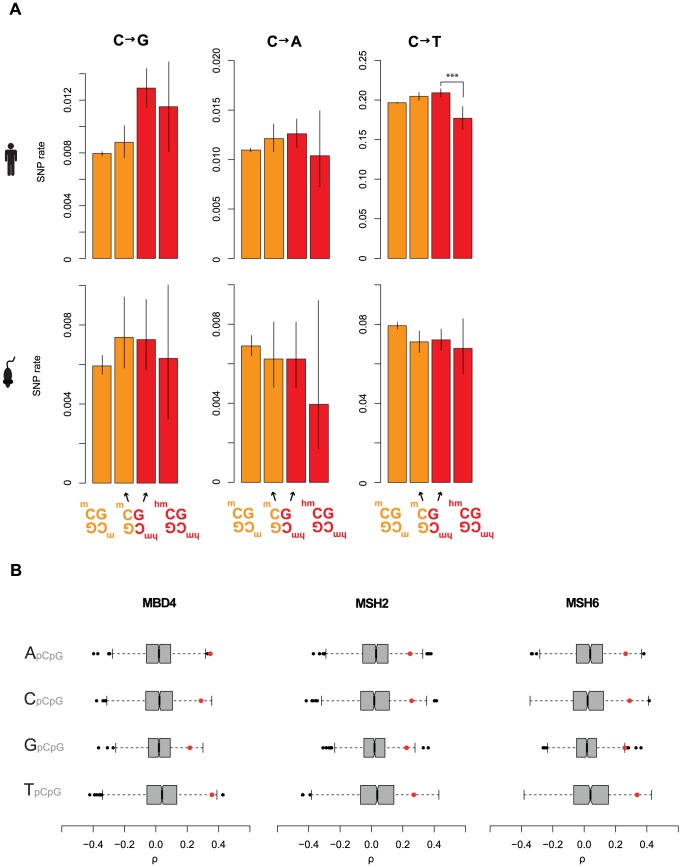
Elevated C to G rates at asymmetrically hydroxymethylated sites. (A) Rates of cytosine loss in the human and mouse population as a function of methylation status and symmetry. Only significant differences between rates at symmetrically and asymmetrically hydroxymethylated 5hmC sites are shown (***P<0.001). Error bars are 95% confidence intervals, calculated using Wilson's interval score for single proportions. Rates for all possible base changes are higher in an asymmetric context for both human and mouse, which is not expected to occur by chance (P<0.04, binomial test). (B) For different upstream contexts, correlation coefficients (Spearman's rho) were computed between C to G rates and expression levels for different MMR components (red dots) and their respective set of control genes (grey/black, see main text). Whiskers extend to approximately 1.5*IQR (interquartile range) below/above the bottom/top quartile of the data, (see R documentation [69] for details).

Yu and colleagues characterized hydroxymethylation as predominantly asymmetric - that is, at CpG dinucleotides where one cytosine showed evidence for hydroxymethylation, the cytosine on the opposite strand typically did not [15]. In contrast, 5mC sites are highly symmetric, with 99% of CpG dinucleotides – when methylated – methylated on both strands [16]. Although 5hmC asymmetry might to some extent be owing to low sequencing depth [20], several high resolution studies now support asymmetric hydroxymethylation as a genuine phenomenon [15], [33], [34]. Indeed, asymmetric hydroxymethylation must occur temporarily given that Tet enzymes oxidize a single 5mC site at a time [35]. We therefore examined SNP rates at symmetrically and asymmetrically hydroxymethylated CpGs. Because this analysis requires consideration of consecutive cytosines on opposite strands, we use the total pool of eligible CpG dinucleotides rather than the matched set employed previously. In both human and mouse, rates of cytosine loss at 5hmC sites appear consistently higher when the 5hmC is found in an asymmetric context (Figure 5A, P<0.04, binomial test, testing for consistency of enrichment across mutations and species). Note that symmetrically hydroxymethylated sites are rare, so our power to detect differences for transversions is limited.

## Discussion

We demonstrate here that hydroxymethylated cytosines in human or mouse ES cells show different patterns of sequence variation and evolution compared to their 5mC-methylated counterparts. They are more likely to give rise to C to G transversions segregating in the population, more frequently associated with C to G substitutions in closely related sister species and exhibit higher rates of C to G mutations in tumors. As rates correlate with quantitative levels of 5hmC, Tet expression/binding, and the presence of 5fC, we suggest that rate differences between 5hmC and 5mC sites – consistently observed across different functional and sequences contexts – are likely mutational in origin and mechanistically linked to hydroxymethylation rather than the result of complex context biases that have escaped detection. Our results also suggest that hydroxymethylation patterns in ES cells are at least in part predictive of hydroxymethylation patterns in an evolutionarily relevant germline context. Neuron-specific 5hmC sites, which should have no bearing on mutation dynamics in the germline, exhibit rates indistinguishable from matched 5mC sites as predicted. Conversely, mESC 5hmC sites overlap more frequently than 5mC sites with regions that are enriched for 5hmC during different stages of spermatogenesis.

The results above are consistent with a model where hydroxymethylation has a causal role in generating higher C to G rates at 5hmC sites. A mutational bias associated with hydroxymethylation might come as a surprise. Several *in vitro* studies concluded that 5hmC correctly templates incorporation of G during replication [36]–[39], in line with results from structural models that DNA polymerases cannot distinguish 5hmC from 5mC [40]. Why then, with replication seemingly unaffected, are 5hmC sites associated with increased transversion rates? One intriguing lead comes from recent *in vitro* evidence that 5caC:G pairs stimulate exonuclease activity of polymerase δ and are bound – as strongly as G:T mismatches – by the mismatch repair (MMR) complex MutSα, which recognizes post-replicative single-base mismatches [39]. Thus, base pairs involving oxidized methylcytosines might be mutagenic despite correctly templating G incorporation if they are (mis-)recognized as lesions by error-prone DNA repair machinery.

That MMR might be implicated in 5hmC-related mutagenesis is intriguing. MMR operates immediately after replication when it needs to discriminate the newly replicated from the template strand, thus exhibiting an intrinsic requirement for asymmetry. In bacteria this requirement is catered for by transient asymmetric methylation [41]. How such guidance is achieved in eukaryotes remains unclear, but it is interesting to speculate that asymmetries in methylation state might affect and perhaps actively coordinate mismatch repair in eukaryotes.

Intriguingly, examining our cancer data, we discovered strong correlations between %C2G and three components of MMR: MSH2, MSH6, and MBD4 (Figure 5B and Table S3). MSH2 and MSH6 form the MutSα heterodimer mentioned above, while MBD4, through an unknown post-transcriptional mechanism, regulates the stability of MSH2, so that MBD4 depletion reduces the number of MMR-competent MutSα complexes [42], [43]. In addition, MBD4 can bind and therefore potentially guide MutSα to methylated and hydroxymethylated CpG sites [44], [45].

It is further worth noting that MSH6 – the MutSα protein that makes direct contact with DNA [46] – was recently identified as one of the very few proteins specifically enriched for binding 5hmC [47]. (Although a related study did not report preferential binding of MSH6 to 5hmCs [45], this might be linked to the nature of the probes employed. Pull-down probes in the former study were made to carry 5hmC via PCR-mediated incorporation of 5hmCTP, an approach expected to lead to 5hmCTP incorporation outside its natural CpG context, thus generating *de facto* asymmetric sites. In contrast, the latter study used a synthetic probe that only contained fully symmetrically hydroxymethylated CpGs (M. Vermeulen, pers. comm.). It therefore seems possible, and consistent with *in vitro* replication studies, which normally only consider a single modified site, that MSH6 might preferentially associate with asymmetrically hydroxymethylated sites. This might explain why asymmetrically hydroxymethylated sites suffer from higher mutation rates, as suggested by Figure 5A).

Based on these observations we suggest the following model that links MMR, hydroxymethylation and elevated C to G transversion rates: 1.) 5hmC can be further oxidized by the Tet family of enzymes to 5fC and 5caC 2.) During DNA replication, 5caC:G pairing induces exonuclease activity of the replicating DNA polymerase δ and is targeted by MutSα [39], either incidentally or as part of a regulated process. 3.) MutSα binding triggers MMR. 4.) G to C transversions are introduced by MMR-affiliated translesion synthesis (TLS) polymerases.

Alternatively, one might consider a slightly more complex model where mutagenic effects derive from an interaction between the MMR and BER DNA repair pathways: After TDG glycosylase removes 5caC/5fC, the resulting abasic site is hijacked by an MMR-affiliated TLS polymerases, leading to elevated transversion rates. An analogous scenario has been suggested for the MSH2- and UNG2-dependent generation of C to G transversion by the TLS polymerase Rev1 in the context of somatic hypermutation [48]–[50]. This model is attractive because it reconciles recent findings of MutSα binding to 5hmC/5caC with known activity of BER at 5caC and 5fC sites. Both models predict MutSα binding to be the rate-limiting factor in the generation of C to G transversions. Detailed biochemical studies will be required to test this hypothesis. However, it is clear from the analyses presented here that hydroxymethlated and methylated CpGs show differential mutation biases that have left a detectable mark on genome evolution, and we propose differences in DNA repair dynamics as a plausible cause of elevated C to G mutation rates at hydroxymethylated cytosines.

## Materials and Methods

### Human methylation data

Starting with all cytosine residues in the human reference genome, we confined analysis to cytosines covered by at least 10 reads in the genome-wide bisulfite sequencing of the H1 hESC cell line conducted by Lister et al [16] (ftp://neomorph.salk.edu/mc/h1_c_basecalls.tar.gz), principally to render results more comparable between species and allow detection of lowly (down to 10%) hydroxymethylated sites. We excluded residues that are part of repeats as annotated in UCSC (hg18) and added information on hydroxymethylation status, assayed at base-resolution for the same cell line [15]. 5hmC, 5mC, and C sites were then defined as described in the main text.

### Human variation data

Data on single nucleotide polymorphisms in the human population from the 1000 Genomes Project [51] were obtained using Ensembl's biomart facility [52] (Ensembl Variation 73; Homo sapiens short variation (GRCh37.p12); 1000 Genomes – All; Validated variations only; Minor allele and frequency). The ancestral allelic state was obtained directly from the 1000 Genomes Project (ftp://ftp.1000genomes.ebi.ac.uk/vol1/ftp/pilot_data/technical/reference/ancestral_alignments). We combined (hydroxy)methylation and polymorphism data after converting all coordinates to hg19 using the liftOver tool [53], and then confined analysis to nucleotides for which the human ancestral state was unambiguous (uppercase residues in the 1000 Genomes Project ancestral alignment) and that were considered assayable by the 1000 Genomes Project (/vol1/ftp/phase1/analysis_results/supporting/accessible_genome_masks/20120824_strict_mask.bed) so as to exclude false negative variation calls.

The Ensembl 6-primate alignment (ftp://ftp.ensembl.org/pub/mnt2/release-75/emf/ensembl-compara/epo_6_primate/) was used to reconstruct substitutions along the chimp lineage. We only considered residues that were cytosines in both human and orang-utan.

### Genotyping H1

The H1 hESC genotype is not the same as the genotype of the human reference genome. This poses the following problem: Bisulfite sequencing works by protecting 5mC residues but not unmethylated cytosines from being converted to uracil. Consequently, whenever sequencing reveals the presence of a U/T that maps to a C in the reference, we would infer that we have recovered an unmethylated C. However, we might also be dealing with a site where the H1 genotype deviates from the reference and is in fact T. In this scenario, erroneously assuming the reference genotype to be present would inflate the number of unmethylated cytosines. This might seem like a minor problem, but can in fact strongly distort downstream evolutionary analysis of unmethylated cytosines, especially when it comes to the analysis of derived allele frequencies (Figure S2).

To be conservative and enable different downstream analyses, we therefore decided to genotype H1 using available H1-derived short read data (RNA-seq, Chip-seq, etc.) from the ENCODE project [54]. Genotype calls were generated from short read alignment files using samtools mpileup and bcftools [55], with parameter values depending on the mapping algorithm used for generating a given short read alignment (see Table S4 for details). Subsequently, we confined analysis to nucleotides covered by at least 20 reads and without a single read suggesting a non-reference genotype. This strict genotype filtering not only resolves the problem inherent in bisulfite sequencing but also ensures that we are dealing with sites that are homozygous in H1. This is important to allow a fair comparison of methylation levels across sites and also facilitates comparison between human and the inbred mouse strains.

### Mouse methylation data

As done for human, we start from a list of all cytosines in the mouse genome (mm9) and subsequently remove nucleotides covered by fewer than 10 reads in the bisulfite sequencing study of the E14TG2a mESC line conducted by [14]. As before, we exclude nucleotides annotated as repeats by UCSC (based on mm9), added hydroxymethylation [15] and converted coordinates to mm10 using liftOver. Unlike Lister et al [16], Stadler and colleagues do not provide binary methylation calls (methylated/unmethylated) for the mESC data. To emulate binary calls in mouse, we examined the distribution of methylation levels for cytosines in a CpG context called methylated/unmethylated in human. Residues where less than 20% of the reads support methylation are predominantly called unmethylated (Figure S1) and we therefore define mouse residues – which follow a similar bimodal distribution of methylation levels overall – as unmethylated/methylated if less/more than 20% of the reads indicate methylation.

### Mouse variation data

In the absence of extensive genome-wide polymorphism data for wild mice populations, we considered polymorphisms across a collection of laboratory mouse strains sequenced by the Sanger Institute [56] (available at http://www.sanger.ac.uk/resources/mouse/genomes/), which are derived from three wild sub-species: *Mus musculus domesticus, Mus musculus musculus, and Mus musculus castaneus* [57]. We used *Mus spretus*, a sister taxon to *Mus musculus* also included in the strain sequencing effort and rat as the outgroup to polarize polymorphisms. Specifically, based on the mouse-rat (mm10-rn5) pairwise alignment from UCSC we retrieved the corresponding *Mus spretus* variants and inferred the base ancestral to all *Mus musculus* strains by parsimony. We only considered sites where genotype calls were made across all strains and further confined analysis to sites where the genotype was congruent between the mouse reference genome and the 129P2/OlaHsd strain from which the mESC line was derived. Note that the bisulfite sequencing study of Stadler et al. [14] explicitly took into account the genotype of the mESC line used and only considered cytosines present in the 129P2/OlaHsd strain, so that we did not have to replicate our H1 pipeline and conduct further genotyping.

### Nucleotide context

Local (±50 nt) and regional (±500 nt) GC content around each eligible cytosine as well as the upstream/downstream neighbouring nucleotides were computed from the reference and reconstructed ancestral sequence for mouse and human. Choice of either ancestral or reference sequence here has no significant impact on the results and we therefore only present data derived from the ancestral sequence.

### Chromatin states and biotypes

Chromatin context is strongly associated with mutation rates in cancer genomes [58] and might also affect mutation dynamics in the germline. At the same time, 5hmC is non-randomly represented across different chromatin states (see main text). To rule out a confounding effect of chromatin environment on C to G transversion biases, we adopted a popular approach to partition genomic regions into mutually exclusive chromatin states based on the distribution of different histone marks and DNA-binding proteins. For H1 hESC, we used pre-existing chromatin state calls from the ENCODE project, where information on the genome-wide distribution of 8 histone modifications (H3K4me1/-me2/-me3, H3K27ac, H3K9ac, H3K36me3, H4K20me1, and H3K27me3) and CTCF binding was used to define 15 chromatin states using the ChromHMM algorithm [59]. For mouse, we collated data on the genome-wide distribution of seven histone marks (H3K4me1/-me2/-me3, H3K36me, H3K9me3, H3K27me3, H4K20me3) in mouse ES cells obtained from two publications [60], [61] and ran ChromHMM to partition the mouse genome into 14 distinct chromatin states. The distribution of H3 histones in [60] was used as input. Coordinates of histone marks were converted to mm10 prior to running ChromHMM. Open chromatin in H1 hESC is defined as per ENCODE (wgEncodeOpenChromSynthH1hescPk.bed). Nucleosome occupancy in mESCs as measured by MNase-Seq was obtained from [62] (GEO accession: GSM945576). Biotypes (exon, intron, intergenic, etc.) were obtained from Ensembl via biomart.

### Cancer genomes

We downloaded aligned short reads for whole genome sequences of 404 cancer samples and paired normal tissues from the cgHub repository of the TCGA project [63]–[65]. We called somatic mutations using Strelka 1.0.5 [66] with default parameters, except for more stringent thresholds for the *bcNoise* and *spanDel* filters (0.05 for both; compared to default values of 0.40 and 0.75, respectively). We excluded mutations in poorly mappable genomic regions (according to a stringent definition in the “CRG Alignability 36” track [67], and the Duke and DAC blacklists from the UCSC browser), as well as the exons of all UCSC genes (+2 intronic nt flanking every exon). Gene expression levels were derived from the TCGA RnaSeqV2 pipeline as transcripts per million (TPM), not transformed or normalized. Only TCGA samples that had both whole-genome mutation data and the RNA-SeqV2 data were considered. Rates were calculated as mutations divided by the number of nucleotides at risk.

To obtain robust estimates of the fraction of mutations in a CpG context, we then confined analysis to 346 cancer samples with at least 1000 inferred single nucleotide variants. When considering individual cancer types, we confined analysis to those with at least 10 sequenced samples.

To establish control gene sets (Figure 4B, 5B) genes were ranked by their median expression across cancer samples and – independently – by their quartile coefficient of dispersion. Genes within ±2000 ranks of the focal gene in both ranked lists were included in the control set (see Table S3 for control set sizes). Defining a more restrictive control set (within ±1000 ranks) yields similar results (not shown). Empirical P values were then simply determined by ranking all correlations and determining the rank of the focal correlation.

We heavily used the bedtools suite for data integration [68].

## Supporting Information

Figure S1 Classifying methylated and unmethylated residues in mouse. The top two panels show the distribution of per-site methylation levels (reads supporting methylation divided by the total number of reads at that site) for cytosines in a CpG context in mouse [14] and human [16], respectively. In the bottom two panels, the human data has been split according to the binary classification into methylated and unmethylated residues provided by [16]. The dotted line at 0.2 highlights the methylation level chosen to classify mouse cytosines into methylated and unmethylated residues.
(EPS)

Figure S2 Derived allele frequencies. Derived allele frequencies (DAFs) in the human population for all eligible 5hmC, 5mC, and C sites and matched 5hmC and 5mC sites. When methylation status is assigned based on mapping bisulfite sequencing reads to the reference genomes (Top panel: Unmatched – reference genotype assumed), there is a striking excess of high frequency derived alleles for unmethylated cytosines. This excess disappears when considering only sites for which the H1 hESC genotype has been confirmed as a cytosine (Middle panel: Unmatched – H1 genotype). This suggests that the excess is caused by alleles where the reference carries the C allele but the cell line (and the majority of the human population) carry the T allele – so that mapping bisulfite reads to the reference would mistakenly indicate the presence of an unmethylated cytosine. The DAF spectra for unmatched but not matched 5mC and 5hmC sites differ significantly from each other (see main text). Note that a similar analysis of DAFs is not appropriate for mouse because the inbred laboratory strains considered here do not constitute a natural evolving population for which allele frequencies would provide a meaningful window into the evolutionary process.
(EPS)

Figure S3 Enrichment of mESC 5hmC residues in 5hmC-enriched regions in the male germline. Gan et al [27] determined 5hmC enrichment during different stages of spermatogenesis at low resolution. Filtering out regions where 5hmC enrichment was detected in their control experiment, we considered the mean enrichment signal at matched sites classified as either 5hmC or 5mC based on (hydroxy)methylation maps in mouse embryonic stem cells (see main text). (A) 5hmC sites show a higher mean enrichment signal than 5mC sites across all stages of spermatogenesis, as expected if ESC-defined 5hmC sites non-randomly reflect 5hmC distribution in the male germline. The difference is more pronounced during earlier stages of spermatogenesis. (B) Comparing C to G SNP rates in regions with and without 5hmC enrichment in developing sperm cells. Significant differences are evident for 5hmC-enriched regions during the SG-B, plpSC, and eST stages (**P<0.01; *P<0.05). Cell types are ordered according to their appearance during spermatogenesis. priSG-A: primitive type A spermatogonia; SG-A: type A spermatogonia; SG-B: type B spermatogonia; plpSC: preleptotene spermatocytes; pacSC: pachytene spermatocytes; rST: round spermatids; eST: elongated spermatids; SZ: spermatozoa. See [27] for details on how these cell types were derived.
(EPS)

Table S1 Matching criteria and ranges for matched pairs analyses.
(DOCX)

Table S2 List of cancer samples classified by cancer subtype.
(TXT)

Table S3 Correlations between the expression of mismatch repair, base excision repair and Tet genes and C to G transversion rates across 346 cancer genomes.
(XLSX)

Table S4 ENCODE data used to genotype H1 hESC.
(DOCX)
