# Supplementary material for: Hydroxymethylated Cytosines Are Associated with Elevated C to G Transversion Rates
Source: PLoS Genet. 2014 Sep 11;10(9):e1004585. doi: 10.1371/journal.pgen.1004585 (PMC4161303; doi:10.1371/journal.pgen.1004585)
Supplement: Table S1 — Matching criteria and ranges for matched pairs analyses. (DOCX) [file pgen.1004585.s004.docx]

**Table S1**

| **Potential confounder** | **Definition** | **Value ranges &**  **matching criteria** |
| --- | --- | --- |
| Local GC content | The fraction of nucleotides in a ±50nt window around the focal site that are either G or C, as applied to the ancestral or reference sequence (see main text) | Theoretical range: 0-100%  Empirical range:   - human: 22-74% - mouse: 23-73%   Matching: nucleotides have to be within the same percentile of GC content |
| Regional GC content | The fraction of nucleotides in a ±500nt window around the focal site that are either G or C, as applied to the ancestral or reference sequence (see main text) | Theoretical range: 0-100%  Empirical range:   - human: 27-69% - mouse: 27-67%   Matching: nucleotides have to be within the same percentile of GC content |
| Chromatin state | As defined by the output of ChromHMM (see Methods) | Human: 14 states (E1-E14)  Note that one state defined in the ENCODE analysis of H1 hESC, which is associated with repetitive sequence, is not represented in our dataset.  Mouse: 14 states (E1-E14)  Matching: nucleotides have to be associated with the same chromatin state. |
| Biotype | Biotypes as defined by Ensembl annotations for mm10 and hg19, respectively. Nucleotides covered by more than one biotype (e.g. being exonic in some transcript isoforms but intronic in others) are excluded | One of exon, intron, 3’UTR, 5’UTR, or intergenic  Matching: nucleotides have to be associated with the same biotype. |
| Upstream nucleotide | The nucleotide immediately upstream on the same strand as the focal cytosine | One of A, C, G, T  Matching: the upstream nucleotide has to be the same type for both partners in the matching pair. |
| Methylation level | Defined by the number of reads supporting a methylated state divided by the number of total reads at that position | Theoretical range: 0-100%  Empirical range: 0-100%  Matching: nucleotides have to be within the same decile of methylation level. |
