# Supplementary material for: Hydroxymethylated Cytosines Are Associated with Elevated C to G Transversion Rates
Source: PLoS Genet. 2014 Sep 11;10(9):e1004585. doi: 10.1371/journal.pgen.1004585 (PMC4161303; doi:10.1371/journal.pgen.1004585)
Supplement: Table S4 — ENCODE data used to genotype H1 hESC. (DOCX) [file pgen.1004585.s007.docx]

**Table S4 -** ENCODE data used to genotype H1 hESC

Source: <http://hgdownload.cse.ucsc.edu/goldenPath/hg19/encodeDCC/>

Data sets used:

wgEncodeBroadHistoneH1hescChd1a301218aStdAlnRep1

wgEncodeBroadHistoneH1hescChd1a301218aStdAlnRep2

wgEncodeBroadHistoneH1hescChd7a301223a1AlnRep1

wgEncodeBroadHistoneH1hescControlStdAlnRep1

wgEncodeBroadHistoneH1hescControlStdAlnRep2

wgEncodeBroadHistoneH1hescCtcfStdAlnRep1

wgEncodeBroadHistoneH1hescCtcfStdAlnRep2

wgEncodeBroadHistoneH1hescEzh239875AlnRep1

wgEncodeBroadHistoneH1hescEzh239875AlnRep2

wgEncodeBroadHistoneH1hescH2azStdAlnRep1

wgEncodeBroadHistoneH1hescH2azStdAlnRep2

wgEncodeBroadHistoneH1hescH3k09me3StdAlnRep1

wgEncodeBroadHistoneH1hescH3k09me3StdAlnRep2

wgEncodeBroadHistoneH1hescH3k27acStdAlnRep1

wgEncodeBroadHistoneH1hescH3k27acStdAlnRep2

wgEncodeBroadHistoneH1hescH3k27me3StdAlnRep1

wgEncodeBroadHistoneH1hescH3k27me3StdAlnRep2

wgEncodeBroadHistoneH1hescH3k36me3StdAlnRep1

wgEncodeBroadHistoneH1hescH3k36me3StdAlnRep2

wgEncodeBroadHistoneH1hescH3k4me1StdAlnRep1

wgEncodeBroadHistoneH1hescH3k4me1StdAlnRep2

wgEncodeBroadHistoneH1hescH3k4me2StdAlnRep1

wgEncodeBroadHistoneH1hescH3k4me2StdAlnRep2

wgEncodeBroadHistoneH1hescH3k4me3StdAlnRep1

wgEncodeBroadHistoneH1hescH3k4me3StdAlnRep2

wgEncodeBroadHistoneH1hescH3k79me2StdAlnRep1

wgEncodeBroadHistoneH1hescH3k79me2StdAlnRep2

wgEncodeBroadHistoneH1hescH3k9acStdAlnRep1

wgEncodeBroadHistoneH1hescH3k9acStdAlnRep2

wgEncodeBroadHistoneH1hescH4k20me1StdAlnRep1

wgEncodeBroadHistoneH1hescH4k20me1StdAlnRep2

wgEncodeBroadHistoneH1hescHdac2a300705aAlnRep1

wgEncodeBroadHistoneH1hescHdac6a301341aAlnRep1

wgEncodeBroadHistoneH1hescJarid1aab26049StdAlnRep1

wgEncodeBroadHistoneH1hescJarid1aab26049StdAlnRep2

wgEncodeBroadHistoneH1hescJmjd2aa300861a1AlnRep1

wgEncodeBroadHistoneH1hescP300kat3bAlnRep1

wgEncodeBroadHistoneH1hescPhf8a301772aAlnRep1

wgEncodeBroadHistoneH1hescPlu1AlnRep1

wgEncodeBroadHistoneH1hescRbbp5a300109aStdAlnRep1

wgEncodeBroadHistoneH1hescRbbp5a300109aStdAlnRep2

wgEncodeBroadHistoneH1hescSap3039731AlnRep1

wgEncodeBroadHistoneH1hescSirt6AlnRep1

wgEncodeBroadHistoneH1hescSuz12051317AlnRep1

wgEncodeCaltechRnaSeqH1hescR1x75dAlignsRep1V2

wgEncodeCaltechRnaSeqH1hescR1x75dAlignsRep2V2

wgEncodeCaltechRnaSeqH1hescR1x75dSplicesRep1V2

wgEncodeCaltechRnaSeqH1hescR1x75dSplicesRep2V2

wgEncodeCaltechRnaSeqH1hescR2x75Il200AlignsRep1V2

wgEncodeCaltechRnaSeqH1hescR2x75Il200AlignsRep2V2

wgEncodeCaltechRnaSeqH1hescR2x75Il200AlignsRep3V2

wgEncodeCaltechRnaSeqH1hescR2x75Il200AlignsRep4V2

wgEncodeCaltechRnaSeqH1hescR2x75Il200SplicesRep1V2

wgEncodeCaltechRnaSeqH1hescR2x75Il200SplicesRep2V2

wgEncodeCaltechRnaSeqH1hescR2x75Il200SplicesRep3V2

wgEncodeCaltechRnaSeqH1hescR2x75Il200SplicesRep4V2

wgEncodeCaltechRnaSeqH1hescR2x75Il400AlignsRep1V2

wgEncodeCaltechRnaSeqH1hescR2x75Il400SplicesRep1V2

wgEncodeCshlLongRnaSeqH1hescCellLongnonpolyaAlnRep1

wgEncodeCshlLongRnaSeqH1hescCellLongnonpolyaAlnRep2

wgEncodeCshlLongRnaSeqH1hescCellPapAlnRep1

wgEncodeCshlLongRnaSeqH1hescCellPapAlnRep2

wgEncodeCshlLongRnaSeqH1hescCytosolLongnonpolyaAlnRep2

wgEncodeCshlLongRnaSeqH1hescCytosolPapAlnRep2

wgEncodeCshlLongRnaSeqH1hescNucleusLongnonpolyaAlnRep2

wgEncodeCshlLongRnaSeqH1hescNucleusPapAlnRep2

wgEncodeCshlShortRnaSeqH1hescCellShorttotalTapAlnRep1

wgEncodeCshlShortRnaSeqH1hescCellShorttotalTapAlnRep2

wgEncodeCshlShortRnaSeqH1hescCytosolShorttotalTapAlnRep2

wgEncodeCshlShortRnaSeqH1hescNucleusShorttotalTapAlnRep2

wgEncodeOpenChromChipH1hescCmycAlnRep1

wgEncodeOpenChromChipH1hescCtcfAlnRep1

wgEncodeOpenChromChipH1hescPol2AlnRep1

wgEncodeOpenChromDnaseH1hescAlnRep1

wgEncodeOpenChromDnaseH1hescAlnRep2

wgEncodeOpenChromFaireH1hescAlnRep1

wgEncodeOpenChromFaireH1hescAlnRep2

wgEncodeRikenCageH1hescCellLongnonpolyaAln

wgEncodeRikenCageH1hescCellPamAln

wgEncodeRikenCageH1hescCellPapAlnRep1

wgEncodeRikenCageH1hescCellPapAlnRep2

wgEncodeRikenCageH1hescCytosolPapAlnRep2

wgEncodeRikenCageH1hescNucleusPapAlnRep2

Processing: Data files were downloaded in .bam format and filtered for mapping quality to only retain “uniquely” mapping reads.

Data from the Broad Institute (mapped with MAQ), Riken (Delve) and the OpenChromChip data (bwa) were filtered for mapping quality >=25

Data from Caltech and CSHL (bowtie) where filtered for mapping quality =255
